# Investigation of the Clinical Value of Four Visualization Modalities for Congenital Heart Disease

**DOI:** 10.3390/jcdd11090278

**Published:** 2024-09-05

**Authors:** Shen-yuan Lee, Andrew Squelch, Zhonghua Sun

**Affiliations:** 1 Discipline of Medical Radiation Science, Curtin Medical School, Curtin University, Perth, WA 6845, Australia; shen-yuan.lee@postgrad.curtin.edu.au; 2 School of Earth and Planetary Sciences, Faculty of Science & Engineering, Curtin University, Perth, WA 6845, Australia; a.squelch@curtin.edu.au; 3 Curtin Health Innovation Research Institute (CHIRI), Curtin University, Perth, WA 6845, Australia

**Keywords:** 3D printing, virtual reality, congenital heart disease, visualization, communication, modality

## Abstract

Diagnosing congenital heart disease (CHD) remains challenging because of its complex morphology. Representing the intricate structures of CHD on conventional two-dimensional flat screens is difficult owing to wide variations in the pathologies. Technological advancements, such as three-dimensional-printed heart models (3DPHMs) and virtual reality (VR), could potentially address the limitations of viewing complex structures using conventional methods. This study aimed to investigate the usefulness and clinical value of four visualization modalities across three different cases of CHD, including ventricular septal defect, double-outlet right ventricle, and tetralogy of Fallot. Seventeen cardiac specialists were invited to participate in this study, which was aimed at assessing the usefulness and clinical value of four visualization modalities, namely, digital imaging and communications in medicine (DICOM) images, 3DPHM, VR, and 3D portable document format (PDF). Out of these modalities, 76.4% of the specialists ranked VR as the best for understanding the spatial associations between cardiac structures and for presurgical planning. Meanwhile, 94.1% ranked 3DPHM as the best modality for communicating with patients and their families. Of the various visualization modalities, VR was the best tool for assessing anatomical locations and vessels, comprehending the spatial relationships between cardiac structures, and presurgical planning. The 3DPHM models were the best tool for medical education as well as communication. In summary, both 3DPHM and VR have their own advantages and outperform the other two modalities, i.e., DICOM images and 3D PDF, in terms of visualizing and managing CHD.

## 1. Introduction

The clinical management of congenital heart disease (CHD) poses significant challenges owing to its diverse morphologies that vary between individuals [1,2,3,4,5]. A comprehensive understanding of anomalous cardiac structures is crucial for successful surgical intervention, when necessary. However, current visualization methods based on cardiac computed tomography or magnetic resonance imaging with volume rendering lack realism as they fail to depict the actual depth of the object. Over the past two decades, three-dimensional (3D) printing has emerged as an important technique in cardiovascular medicine to demonstrate the geometric associations between intra- and extra-cardiac structures [6,7,8,9,10]. Recent research has established the utility of 3D-printed heart models (3DPHMs) in aiding surgical decision making in cases where conventional imaging provides inconclusive results [11]. However, despite the benefits in surgical planning, training, and education, the time and cost of producing 3DPHMs hinder their widespread application in the medical field [12]. Moreover, standardization and quality control processes are required because of the novelty of the technology in medical settings [13].

Virtual reality (VR) has transformed the medical field by offering innovative solutions for both education and patient care [14,15,16]. VR technology is making a groundbreaking impact in the management and understanding of CHD [17,18,19,20]. By creating detailed, immersive 3D heart and vascular models, VR allows cardiologists and cardiac surgeons to explore the unique anatomical complexities of each patient’s heart condition in a way that was previously not possible with conventional imaging methods [21,22,23]. This enhanced visualization aids in accurate diagnosis, surgical planning, and simulation, enabling medical professionals to visualize the heart’s structures and plan interventions with unprecedented precision [24]. For educational purposes, VR serves as an invaluable tool for training medical students and professionals, providing them with a hands-on experience of congenital heart defects without the need for invasive procedures. In addition, VR facilitates an enhanced understanding of CHD by patients and their families, thus fostering clear communication about the condition, treatment options, and expected outcomes. By leveraging the capabilities of VR, healthcare providers can improve surgical outcomes, reduce the risks associated with complex procedures, and enhance the quality of life for patients with CHD [25].

The introduction of 3D portable document format (PDF) technology in the medical field has revolutionized the sharing and visualization of medical information, offering considerable advantages in diagnostics, patient education, and collaborative care [26]. Unlike conventional two-dimensional images, 3D PDFs permit the interactive visualization of complex anatomical structures directly within a PDF document. Therefore, healthcare professionals are able to manipulate and examine patient-specific models in three dimensions.

This study aimed to investigate the usefulness and clinical value of four visualization modalities in three different cases of CHD, including ventricular septal defect (VSD), double-outlet right ventricle (DORV), and tetralogy of Fallot (ToF). The rationale of choosing these three case scenarios is that VSD represents a simple CHD condition, while DORV and ToF represent moderately complex CHD situations; thus, this allows us to determine the clinical value of these visualization modalities in assessing different congenital heart defects. The four visualization modalities comprised DICOM (digital imaging and communications in medicine) images, 3DPHM, 3D VR, and 3D PDF. To determine the clinical value of these four visualization modalities, cardiologists and cardiac surgeons were invited to compare their usefulness in presurgical planning, medical education, and communication with patients and their families.

## 2. Materials and Methods

### 2.1. Generation of Digital Heart Models

Anonymized DICOM formats of cardiac computed tomography angiography (CCTA) images with simple and complex CHD conditions were collected in this study. The Mimics Innovation Suite 22.0 (Materialise, Leuven, Belgium) was used for the postprocessing and segmentation of the images. DICOM images were imported into Mimics for segmentation based on thresholding and editing across multiple slices. Voxels within a certain range of Hounsfield units (HU) were isolated via thresholding by selecting different threshold ranges, allowing the creation of different masks to depict various anatomical features. The heart’s blood pool was separated from other structures, with a 2 mm thickness layer applied externally to assist in the heart model’s printing process. The 3-Matic software 22.0 (Materialise, Leuven, Belgium) was utilized to repair holes in the digital model using wrap and smooth functions. The standard tessellation language (STL) file was then transferred to Meshmixer 3.5 (Autodesk, San Rafael, CA, USA) for additional modifications. The 3D model was split into a two-part component to inspect the heart’s internal structure and provide a clear view and assessment of each model’s specific defect.

### 2.2. 3D Printing

**Figure 1:**
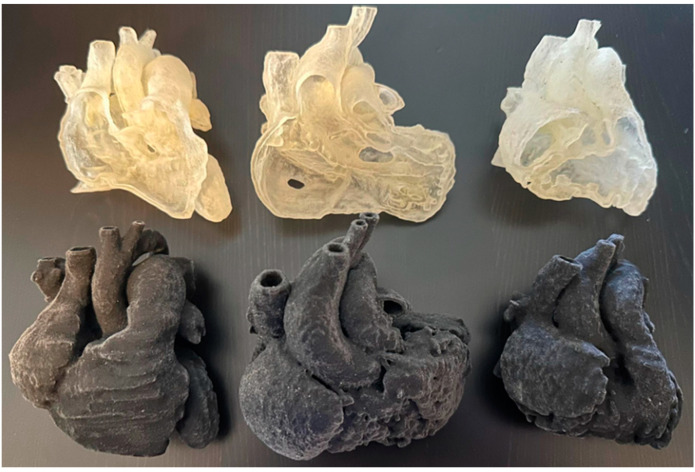
Models were printed with a flexible material, Agilus 30 (Objective 3D, Stratasys, Melbourne, VIC, Australia). VSD in the left column, DORV in the middle column, and ToF in the right column.

STL files were transferred into Objet500 Connex3 (Objective 3D, Stratasys, Melbourne, VIC, Australia) for printing. The models were printed with a flexible material, Agilus 30 (Objective 3D, Stratasys, Melbourne, VIC, Australia), which has a shore hardness of 30 A (materials with softer, more flexible features similar to those of human tissues) in both clear and black color. The models were printed in two ways, as depicted in Figure 1. The cost of the whole heart model was approximately 350 AUD each and that of the plane-cut model was approximately 260 AUD each. The printing time was approximately 5 h for each model.

### 2.3. VR

**Figure 2:**
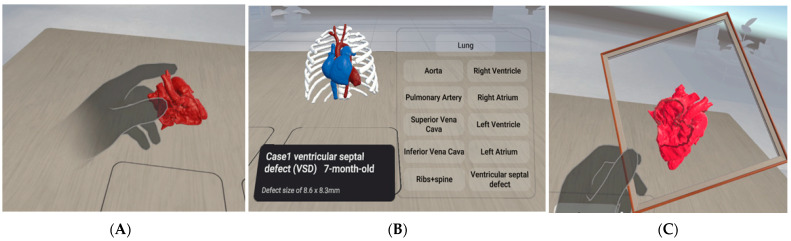
The three functions in VR visualization: (**A**) grabbing the model; (**B**) turning body parts on/off; and (**C**) plane-cutting of the heart.

Three-dimensional VR models were built using Unity 3D (Unity Technologies, San Francisco, CA, USA) with C# coding for additional functionality, such as for grabbing. Creating a VR project for the Oculus Quest 2 headset in the context of CHD using Unity involves several key steps to ensure an immersive and educational experience. Initially, the developers must install Unity with the appropriate VR development support packages and the Oculus Integration package from the Unity Asset Store, which includes essential tools, scripts, and prefabs for Oculus VR development. The digital heart model STL file can be imported using advanced 3D modeling software, Meshmixer 3.5 (Autodesk, San Rafael, CA, USA) and is then imported into Unity. Within Unity, script interactions and animations to simulate various heart conditions using VR hand tracking allow users to manipulate the view or interact with the model to better understand the heart’s structure and function. Implementing user interface elements that provide educational content, such as turning on and off the body part and performing a live plane-cut of the heart, can enhance learning. To ensure a smooth and immersive experience, the VR environment for Oculus Quest 2 must be optimized by considering its processing capabilities to maintain high performance and visual quality. The VR project, including its three main functions, is shown in Figure 2. A video clip demonstrating how VR works in this study is presented in Supplementary File S1.

### 2.4. 3D PDF

**Figure 3:**
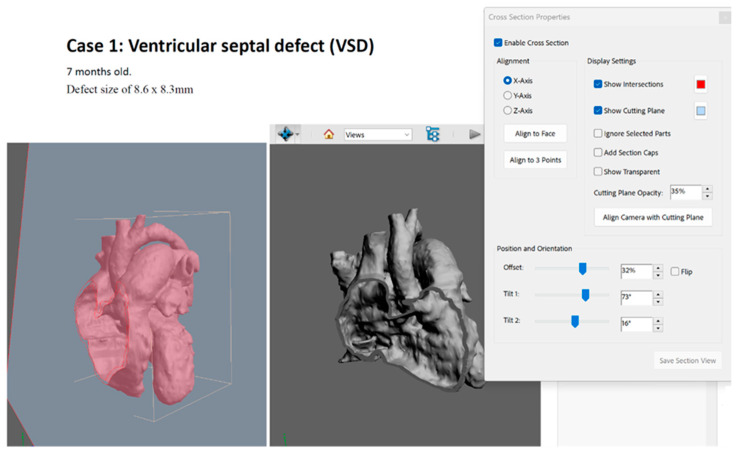
Three-dimensional PDF of the patient with VSD. The segmented volume data file in STL was used to generated the 3D PDF visualization.

Adobe Acrobat Pro (Adobe Systems Incorporated, San Jose, CA, USA) was used to create 3D PDF for the three cases of CHD. The STL file of each case was imported into the PDF file with the case’s details (Figure 3), for both the whole heart and the plane-cut heart. The “Add Default Views” function was used to select the view that would allow the participant to visualize the defect in each case. The cross-sectional property function was used to immediately display the cross-section of the 3D model, cutting it in half and looking inside, to determine the axis (*x*, *y*, or *z*) with which the cross-section was aligned.

### 2.5. Participant Recruitment and Data Collection

**Figure 4:**
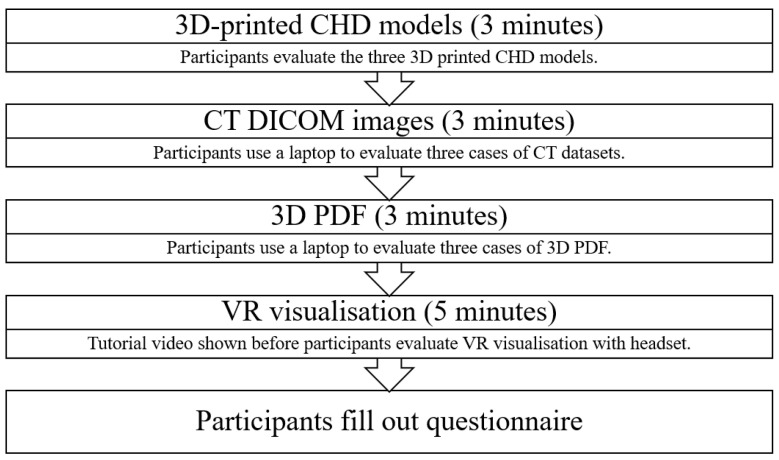
Process for each participant to visualize the 4 modalities and provide their ranking scores.

Seventeen cardiac professionals were recruited for this study from public and private hospitals in Western Australia. Each participant was involved in comparing and ranking the usefulness of the four visualization modalities across three different CHD cases, which took approximately 20 min (Figure 4). The 3DPHMs were printed using the flexible material Agilus30. The DICOM images and 3D PDFs were displayed on a 2D flat screen, whereas the 3D VR experiences were shown via a VR headset. An open-source DICOM viewer, RadiAnt (Medixant, Poznan, Poland), was used by the participants to view the DICOM images of each case on a laptop. Before evaluating the VR project, a tutorial video was shown to provide a better understanding of how to operate it with the VR headset. Questionnaires were distributed after demonstrating the four modalities, with questions based on ranking the usefulness in three major areas, namely, presurgical planning, medical education, and communication with patients and their families. The questionnaire is provided in Supplementary File S2. Each modality was ranked for each case, with 1 indicating the best and 4 indicating the worst modality.

### 2.6. Statistical Analysis

Quantitative data from the questionnaire were analyzed using the IBM SPSS statistical package, version 26 (IBM Corp, Armonk, NY, USA). The normality of the data was evaluated using a normal probability plot, and skewness and kurtosis of the distribution were reported. A *p*-value of <0.05 was considered statistically significant.

## 3. Results

In total, 17 participants were included in this study, of which 12 were men and 11 had 10–20 years of working experience. There were 3 cardiac surgeons, 13 cardiologists, and 1 radiologist (Table 1).

The mean rank of each modality for each question is presented in Table 2. The participants were requested to rank the modalities from 1 to 4, with 1 indicating the highest preference. Hence, a mean rank approaching 1 signified a favorable perception of the modality by the participants.

VR was the best modality for assessing the anatomical location and vessels. This method exhibited the lowest mean score across all heart defects, indicating that it provides the most accurate evaluation. The 3DPHM and DICOM modalities followed, with 3D PDF being the least effective. Furthermore, VR ranked the highest in spatial association among cardiac structures, with the lowest mean scores, which signified its superior performance in illustrating spatial relationships. The 3DPHM method was the most effective modality for visualizing heart defects, with the lowest mean scores. This method was followed by VR, DICOM, and 3D PDF. In learning about pathology, 3DPHM outperformed the other modalities, offering the clearest insights into pathology, with the lowest mean scores.

VR was the best presurgical tool, as inferred from its lowest mean scores. The 3DPHM, DICOM, and 3D PDF modalities came next, with 3D PDF being the least preferred. For medical education, 3DPHM and VR were highly effective, and both showed lower mean scores than DICOM and 3D PDF, with 3DPHM slightly surpassing the others in certain cases. The 3DPHM modality stood out as the best communication tool, with significantly lower mean scores, indicating its effectiveness.

The quality of visualization was quantified by the mean score, with a rating scale from 1 (well visualized) to 4 (not visualized), and the standard deviation (SD) implied the variability in the scores (Table 3). Across all anatomical structures, VR consistently showed excellent visualization capabilities, with low mean scores and minimal variability. DICOM was also effective, particularly for the aorta and pulmonary artery. The 3DPHMperformed well but was not as consistent as VR. Three-dimensional PDF was the least effective modality, especially for complex visualizations such as defects.

Table 4 presents the mean scores of the different modalities for each question. The participants were requested to rate the modalities on a scale of 1–10 for each question, with 10 being the highest score. For presurgical planning, VR was rated as the most useful modality, with a mean score of 8.71 and an SD of 1.1. Thus, this method appears to have a high average usefulness, with moderate variability in the responses. 3DPHMfollowed closely, with a mean of 8.47 and a slightly lower SD of 1.07, alluding a similarly high level of usefulness but with slightly more consistent responses among the participants. In addition, DICOM demonstrated considerable usefulness, with a mean of 7.82 and the lowest SD of 0.95, indicating a more uniform perception among the respondents. Three-dimensional PDFs scored significantly lower in usefulness for presurgical planning, with a mean of 5.25 and the highest SD of 1.41, which implies both a lower perceived usefulness and a higher variability in the responses. In the context of educational tools for medical students or junior doctors, VR scored the highest at 9.12, which indicates its effectiveness in providing immersive learning experiences. The 3DPHM modality also scored highly at 8.94, emphasizing its value in tangible learning aids. DICOM, while still useful, scored lower at 7.18, and 3D PDFs were rated the least useful at 4.65, reflecting potentially limited engagement or interactivity compared to the other modalities.

Overall, VR and 3DPHM were consistently valued across both categories for their high engagement and effectiveness in medical education and planning, with VR having a slight advantage. Conversely, 3D PDFs were consistently rated as the least useful modality, which shows that more immersive or tangible technologies are preferred in these contexts. There were no significant differences in the scores given to these modalities under the three different CHD case scenarios.

**Figure 5:**
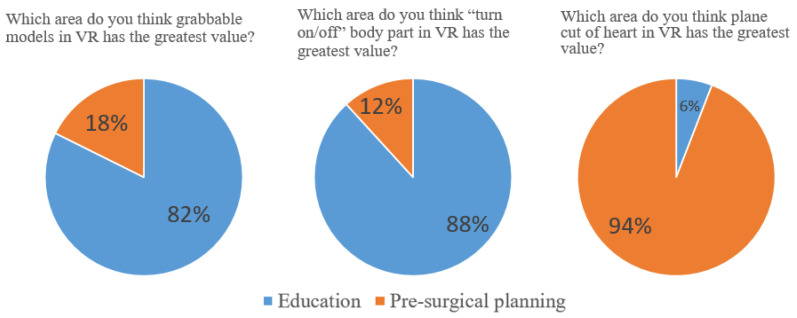
Responses from the participants regarding the usefulness of VR functions.

Of the 17 participants, 14 (82%) and 15(88%) opined that the grabbing function and turn on/off option for the body parts in VR exhibited the greatest value in the education area, respectively. Moreover, 16 (94%) indicated that the live plane-cut function displayed the greatest value in the presurgical planning area (Figure 5). None of the participants provided “no” as the response to all three questions, which suggests the positive inclination of the participants toward this aspect.

## 4. Discussion

The results of this study demonstrated the consistent superiority of 3DPHM and VR over 3D PDFs and DICOM in almost all evaluated categories. Feedback from cardiac specialists and physicians regarding the three CHD cases indicated promising outcomes for the four visualization modalities. VR emerged as the leading modality in assessing anatomical locations and vessels across all CHD types, implying its strength in providing clear and detailed visualizations for the accurate identification and understanding of complex heart structures. For comprehending the spatial associations between cardiac structures, VR exhibited superior performance, which showed its capability to offer an immersive experience that can augment the understanding of intricate spatial dynamics within the heart, which is crucial for both diagnostics and surgical planning.

Both VR and 3DPHM performed well in facilitating pathology learning, with VR having a slight edge. This finding highlights the potential of these techniques in medical education, providing interactive and engaging methods for studying and comprehending CHD pathologies. VR proved to be the most effective presurgical planning tool, which emphasizes its value in presurgical preparations owing to the benefit of 3D visualization and simulation. This capability could considerably influence surgical outcomes by enabling the use of precise and informed surgical strategies. In medical education, 3DPHM and VR showed immense promise, with 3DPHM slightly outperforming VR. The 3DPHM modality was the best communication tool, which indicates that physical models are particularly effective in explaining complex cardiac conditions to patients and their families. This modality can enhance patient understanding and involvement in the care process. This is consistent with similar recent reports, highlighting the usefulness of VR and 3DPHM in CHD [16,24].

The advent of 3D printing technology has substantially transformed the landscape of CHD management and has offered innovative approaches for diagnosis, surgical planning, medical education, and patient communication. In their study, Valverde et al. demonstrated that 3D-printed models of complex CHD cases facilitated an in-depth understanding of anatomical structures, thereby aiding in the identification of optimal surgical pathways and potential complications. These models enable surgeons to simulate and rehearse procedures, which can potentially reduce operative times and improve surgical precision, with surgical decisions being modified in 48% of CHD cases after the use of 3D-printed models [27]. Similarly, Kiraly et al. reported their multidisciplinary team experience of using 3D-printed models in managing complex CHD scenarios [23]. Personalized 3D-printed models were developed in 15 cases of patients with complex congenital cardiac defects, and the 3D-printed models were found to refine diagnosis in 13 cases and provide new information in 9 cases. With the aid of the 3D-printed models, intracardiac repair was modified in 13 cases.

The 3DPHM modality allows students and medical professionals to physically explore cardiac defects, which fosters a more intuitive understanding of congenital anomalies compared to conventional 2D imaging techniques. This interactive learning approach can improve knowledge retention and clinical skills among learners [28]. Furthermore, 3DPHM models have emerged as a powerful tool for patient education and communication. Healthcare providers can more effectively explain the nature of the disease, the proposed surgical interventions, and the expected outcomes to the patients and their families with the aid of physical models of heart defects. This approach not only enhances patient understanding but also facilitates informed decision making and augments patient satisfaction with the provided care [29]. The findings from this investigation suggest that 3DPHM is the best modality for communication with patients and their family.

The ability of VR technology to enhance diagnostic accuracy and surgical planning in CHD has been increasingly recognized. VR enables clinicians to visualize complex cardiac anomalies in ways that were hitherto not possible with standard 2D imaging techniques. Studies by Priya et al. demonstrated that VR-assisted preoperative planning could significantly improve the understanding of anatomical structures, thus aiding in the strategic planning of surgical interventions to potentially reduce operative times and improve patient outcomes [22]. Kieu et al. established the role of VR in enhancing the educational experience of medical students and professionals. Thus, VR can facilitate a more intuitive grasp of spatial relationships and surgical techniques, which can enhance the learning outcomes [17]. A study by Lim et al. reported that VR-based patient education can lead to higher satisfaction rates and a better understanding of medical conditions and treatments [25]. Moreover, studies documented the use of VR for preoperative planning, which allowed surgeons to explore patient-specific anatomy and devise surgical strategies for more precise and effective interventions [30]. However, the integration of VR in CHD care has its own limitations. Technical limitations, cost implications, and the need for specialized training are some of the factors that prevent its widespread adoption. In addition, there is a growing need for standardized protocols and validation studies to ensure the reliability and accuracy of VR models. In our study, VR was considered the best modality for presurgical planning as the live plane-cut function allows cardiac professionals to cut open the heart in any angle.

The findings of Lau et al. are similar to ours, with mixed-reality models ranking the best in preoperative planning and 3DPHM being the preferred tool in communication with patients [24]. The difference between the two studies is that our research compared 3D PDF with three other visualization modalities. Furthermore, the headset and VR environment and function were different. While Oculus Quest 2 was used in this study, HoloLens 2 was utilized in Lau’s study.

One of main advantages of VR over the other three modalities lies in its ability to provide a 3D immersive environment, which significantly enhances user understanding of complex anatomical structures. This is especially important when dealing with complex congenital heart defects due to a wide range of complexity associated with different CHD conditions. VR was ranked the best tool for providing realistic 3D visualization of the spatial relationship between cardiac structures and defects; therefore, it plays an important role in guiding the presurgical planning of CHD surgeries, with potential reductions in the risks or complications that may arise from the challenging surgery procedures. This is consistent with a recent study comparing VR with 3D printing and 3D PDF in imaging patients with CHD [31]. Raimondi et al. compared VR models with 3D-printed models and 3D PDF in three different types of CHD conditions with regard to their performance in visualizing anatomical structures and congenital heart defects. VR was ranked to be advantageous in the following, compared to the other two 3D visualization modalities: demonstrating anatomical structures, easy navigation, faster, reproducible, and less prone to human errors.

The use of VR in CHD surgery or surgical planning is not well studied in the current literature. Priya and colleagues reported the application of VR for baffle planning in two CHD cases [22]. Their developed VR interface model allowed surgeons to simulate different baffle configurations, enhancing the baffle surgical planning process and the patient outcomes. Despite findings based on a few cases, these studies show that VR is a promising approach in CHD visualization and surgical planning. The results of our study also confirm these case reports regarding the potential value of VR in CHD surgical planning. Although our analysis is subjective due to its qualitative nature, it does provide insights into the participants (cardiac specialists)’ experiences, perceptions, or opinions on the usefulness of VR versus other 3D modalities in CHD management. Thus, our findings lay a good foundation for further research on the utility of VR in guiding CHD surgical planning, in addition to its value in improving communication and comprehension among multidisciplinary teams. Furthermore, VR models allow cardiac surgeons to perform the real-time exploration of cardiac anatomy compared to physical and static 3D-printed models, thus assisting clinical decision making.

Another advantage of VR technology is its cost effectiveness and time efficiency compared to 3DPHM. In this study, we used an Oculus Quest 2 headset for the VR demonstration, which cost around 400–500 AUD. In contrast, each 3D-printed heart model cost about 300 AUD, with a total of 900 AUD for the three models in this study. Another limitation of using 3D printing technology is the time spent on printing and the cleaning process, which take much longer than VR visualization.

The main advantage of 3DPHM is that it provides a physical model for hands-on learning and training experience. Thus, it serves as a useful tool for medical education and clinical communication as 3D-printed personalized physical models provide direct views of the anatomical structures to enhance the learning experience and communication with patients or families, as well as clinical colleagues [32,33].

This study has certain limitations. First, the allocated time might have been insufficient for a complete evaluation of the modality, which might have potentially led to biased responses from the participants. Furthermore, we did not randomize the order of these modalities when presenting them to the participants; thus, we could not avoid the potential risk of biased opinions by the participants when ranking these modalities. This should be considered in future studies. Second, despite receiving a brief tutorial on the use of 3D PDFs, the participants faced challenges in navigating the 3D heart models on a flat 2D screen. Finally, the number of participants was small, and a sufficient number of cardiac surgeons were not available to assess the clinical value of these modalities. Future studies should seek opinions from more cardiac surgeons to further quantify these benefits and potentially explore the advantage of these tools in other areas of medical diagnosis and treatment for enhancing patient care. A further follow-up study is desirable to include two aspects that will further strengthen our study’s outcomes: the inclusion of patients and their relatives, giving opinions on which modality helped them understand their condition best, and surgeons, giving their views on whether they changed their approach or strategy following a review of the case using these modalities. This will allow us to better understand how the selection of these modalities will change current practices and benefit patient care when planning CHD surgeries.

## 5. Conclusions

The findings from this study have established that, out of the four visualization modalities, VR is the best tool for assessing anatomical location and vessels, understanding the spatial relationships between cardiac structures, and conducting presurgical planning. The 3DPHM models are the best tool for medical education and communication. For the comprehensive and effective management of CHD, 3DPHM and VR are superior modalities, offering detailed and interactive ways to comprehend and address complex cardiac conditions. These tools hold immense promise for augmenting the outcomes in medical education, surgical planning, and patient communication.

## Figures and Tables

**Table 1:** Characteristics of the study participants.

Variables	No. of Participants (%)
Sex	
Male	12 (70.6)
Female	5 (29.4)
Working Experience (years)	
Below 10	3 (17.6)
10 to 20	11 (64.8)
Above 20	3 (17.6)
Occupation	
Cardiac surgeon	3 (17.6)
Cardiologist	13 (76.5)
Radiologist	1 (5.9)

**Table 2:** The mean ranks of different modalities for each question.

Question	Modality	Ventricular Septal Defect			Double-Outlet Right Ventricle			Tetralogy of Fallot		
		Mean	SD	*p*-Value	Mean	SD	*p*-Value	Mean	SD	*p*-Value
Assessment of anatomicallocation and vessels	3DPHM	1.82	0.64	0.05	2.18	0.53	0.01	2.17	0.53	0.05
	VR	1.65	0.79		1.68	0.86		1.64	0.93	
	3D PDF	3.59	0.62		3.71	0.77		3.71	0.59	
	DICOM	2.82	1.19		2.47	1.12		2.47	1.17	
Spatial relationship between the cardiac structures	3DPHM	1.76	0.66	0.00	1.94	0.66	0.02	1.94	0.66	0.01
	VR	1.53	0.71		1.41	0.71		1.59	0.93	
	3D PDF	3.41	0.79		3.47	0.79		3.47	0.62	
	DICOM	3.29	0.77		3.18	0.81		3.12	1.06	
Visualize the heart defects	3DPHM	1.53	0.51	0.05	1.94	0.56	0.05	1.94	0.66	0.05
	VR	1.89	0.78		1.71	0.85		1.82	0.95	
	3D PDF	3.59	0.62		3.47	0.79		3.53	0.62	
	DICOM	2.94	1.09		2.88	1.22		2.71	1.26	
Learn about the pathology	3DPHM	1.65	0.51	0.00	1.71	0.59	0.01	1.76	0.56	0.01
	VR	1.59	0.79		1.53	0.72		1.65	0.93	
	3D PDF	3.41	0.62		3.53	0.62		3.68	0.61	
	DICOM	3.35	0.87		3.24	0.83		2.94	0.89	
Presurgical tool	3DPHM	2.18	0.53	0.00	1.82	0.53	0.03	1.88	0.49	0.03
	VR	1.41	0.71		1.35	0.71		1.47	0.94	
	3D PDF	3.59	0.87		3.71	0.59		3.65	0.61	
	DICOM	2.82	1.01		3.11	0.61		3.11	0.79	
Medical education	3DPHM	1.59	0.51	0.01	1.47	0.51	0.03	1.59	0.51	0.03
	VR	1.53	0.72		1.76	0.75		1.71	0.92	
	3D PDF	3.35	0.71		3.29	0.69		3.41	0.62	
	DICOM	3.53	0.53		3.47	0.79		3.29	0.85	
Communication tools	3DPHM	1.06	0.24	0.00	1.06	0.24	0.00	1.06	0.24	0.00
	VR	2.76	0.67		2.82	0.64		2.71	0.69	
	3D PDF	2.52	0.87		2.47	0.81		2.59	0.87	
	DICOM	3.65	0.61		3.71	0.59		3.65	0.61	
Reduce errors during the surgery	3DPHM	2.01	0.78	0.36	1.94	0.66	0.85	2.17	0.6	0.92
	VR	1.98	0.92		2.01	0.87		1.95	0.99	
	3D PDF	2.98	0.56		2.88	0.75		2.59	0.61	
	DICOM	2.65	0.86		2.65	0.79		2.82	0.95	

**Table 3:** The mean score of visualization of the anatomical locations.

Location	Modality	Mean	SD
Heart chamber	3DPHM	1.65	0.61
	VR	1.12	0.33
	3D PDF	2.06	0.65
	DICOM	1.29	0.47
Aorta	3DPHM	1.18	0.39
	VR	1.12	0.33
	3D PDF	1.41	0.62
	DICOM	1.05	0.24
Pulmonary artery	3DPHM	1.12	0.33
	VR	1.06	0.24
	3D PDF	1.41	0.61
	DICOM	1.06	0.24
Defect	3DPHM	1.29	0.47
	VR	1.18	0.39
	3D PDF	2.24	0.75
	DICOM	1.76	0.83

A rating scale of 1 = well visualized; 2 = visualized; 3 = poorly visualized; and 4 = not visualized was used.

**Table 4:** The mean scores of different modalities for each question.

Question	Modality	Mean	SD
Usefulness for presurgical planning	3DPHM	8.47	1.07
	VR	8.71	1.1
	3D PDF	5.25	1.41
	DICOM	7.82	0.95
Usefulness of educational tools for medical students or junior doctors	3DPHM	8.94	0.83
	VR	9.12	1.11
	3D PDF	4.65	1.77
	DICOM	7.18	0.88

## Data Availability

Data are contained within the article and Supplementary Materials.

## Supplementary Materials

The following supporting information can be downloaded at https://www.mdpi.com/article/10.3390/jcdd11090278/s1: File S1: Video clip of VR; and File S2: Questionnaire.
