# Supplementary material for: Investigation of the Clinical Value of Four Visualization Modalities for Congenital Heart Disease
Source: J Cardiovasc Dev Dis. 2024 Sep 5;11(9):278. doi: 10.3390/jcdd11090278 (PMC11431982; doi:10.3390/jcdd11090278)
Supplement: Supplementary file 1 [file jcdd-11-00278-s001.zip › jcdd-3061445-supplementary file S2.pdf]

Clinical details

1. What is your position in the hospital?

   ☐ Cardiologist ☐ Cardiac Surgeon ☐ Other__________________

2. How many years have you worked in your professional field?

   ____

Please compare and rank each modality in each of the following cases with 1 being the best, and 4 being the least:

Questions	Case 1 Ventricular Septal Defect				Case 2 Double Outlet Right Ventricle				Case 3 Tetralogy of Fallot			
	3DP models	VR	3D PDF	DICOM images	3DP models	VR	3D PDF	DICOM images	3DP model	VR	3D PDF	DICOM images
3. Can clearly understand the anatomical location and vessels.												
4. Can clearly understand the spatial relationship between the cardiac structures.												
5. Can clearly visualize the heart defects.												
6. It can be used to learn about the pathology.												
7. It can be used as a pre-surgical tool.												
8. It can be used as teaching tools for medical education.												
9. It can be used as a tool to communicate with patient and patient's family about the pathology.												
10. It can help to reduce the error during the surgery.												

11. Visualization of anatomical locations, please rate in each modality. (1= well visualized, 2= visualized, 3= poorly visualized, 4=non-visualized,)

	Locations	3D printed models	VR	3D PDF	DICOM images
1	Heart Chambers				
2	Aorta				
3	Pulmonary arteries				
4	Defect				

12. Please rate the usefulness of each modality in preoperative planning.

	0	1	2	3	4	5	6	7	8	9	10
3D printed models											
VR											
3D PDF											
DICOM images											

13. Please rate the usefulness of each modality in educational tools for medical students or junior doctors about congenital heart disease.

	0	1	2	3	4	5	6	7	8	9	10
3D printed models											
VR											
3D PDF											
DICOM images											

14. How do you feel about the function present in VR? Which medical area do you think it has the greatest value?

Grabbable models ☐ Educational ☐ Pre-surgical planning ☐ Other________________________

Turn on/off body parts ☐ Educational ☐ Pre-surgical planning ☐ Other________________________

Plane cut of heart ☐ Educational ☐ Pre-surgical planning ☐ Other________________________

Additional comments:

15. Additional comments about the advantages or disadvantages of the 3D printed models, VR and 3D PDF compared to DICOM images.
